# Viscosol Treatment Ameliorates Insulin-Mediated Regulation of Dyslipidemia, Hepatic Steatosis, and Lipid Metabolism by Targeting PTP1B in Type-2 Diabetic Mice Model

**DOI:** 10.1155/ije/3914332

**Published:** 2024-12-27

**Authors:** Idrees Raza, Aamir Sohail, Hamza Muneer, Hajra Fayyaz, Zia Uddin, Amany I. Almars, Waheeb S. Aggad, Hailah M. Almohaimeed, Imran Ullah

**Affiliations:** ^1^Department of Biochemistry, Faculty of Biological Sciences, Quaid-i-Azam University, Islamabad 45320, Pakistan; ^2^Department of Biochemistry & Biotechnology, FVAS, Muhammad Nawaz Shareef University of Agriculture, Multan, Pakistan; ^3^Department of Pharmacy, COMSATS University Islamabad, Abbottabad Campus, Abbottabad 22060, Khyber Pakhtunkhwa, Pakistan; ^4^Department of Medical Laboratory Sciences, Faculty of Applied Medical Science, King Abdulaziz University, Jeddah 21589, Saudi Arabia; ^5^Division of Anatomy, Department of Basic Medical Sciences, College of Medicine, University of Jeddah, P.O. Box 8304, Jeddah 23234, Saudi Arabia; ^6^Department of Basic Science, College of Medicine, Princess Nourah bint Abdulrahman University, P.O. Box 84428, Riyadh 11671, Saudi Arabia

**Keywords:** *Dodonaea viscosa*, dyslipidemia, hepatic steatosis, insulin resistance, PTP1B, STZ-HFD mice model, type 2 diabetes

## Abstract

Type 2 diabetes mellitus (T2DM), a metabolic disorder, has the hallmarks of persistent hyperglycemia, insulin resistance, and dyslipidemia. Protein-tyrosine phosphatase 1B (PTP1B) was found to be overexpressed in many tissues in the case of T2DM and involved in the negative regulation of insulin signaling. So, PTP1B inhibition can act as a therapeutic target for T2DM. Numerous studies claimed the anti-inflammatory, hypoglycemic, hepatoprotective, and hypolipidemic activities of *Dodonaea viscosa*. Previously, we generated the high-fat diet (HFD)-low dose streptozotocin (STZ)–induced diabetic male mice model and treated it with a PTP1B inhibitor (5, 7-dihydroxy-3, 6-dimethoxy-2- (4-methoxy-3- (3-methyl-2-enyl) phenyl)-4H-chromen-4-one), isolated from Dodonaea viscosa. In the current study, we aimed to investigate the De novo lipogenesis, adipocyte differentiation, augmentation of lipoproteins clearance, fatty acid uptake, antilipolysis activity, and hepatic steatosis of PTP1B inhibition in adipose and liver tissues of the HFD-STZ–induced diabetic mice model. We found the retrieval of normal morphology of adipocytes and hepatocytes in the compound-treated group. The biochemical parameters showed the gradual reduction of LDL, VLDL, TC, and TG in the serum of the compound-treated group. To further test our hypothesis, real-time PCR was performed, and data revealed the reduction of PTP1B and other inflammatory markers in both tissues, showing enhanced expression of insulin signaling markers (INSR, IRS1, IRS2, and PI3K). Our compound upregulated the adipogenic (PPAR*γ*), lipogenic (SREBP1c, FAS, ACC, and DGAT2), lipoprotein clearance (LPL, LDLR, and VLDLR), fatty acid uptake (CD36 and FATP1), and lipid droplet forming (FSP27 and perilipin-1) markers expressions in adipocytes and downregulated in hepatocytes. Furthermore, we found elevated cholesterol efflux (in adipose and liver) and decreased lipolysis in adipocytes and elevated in hepatocytes. Hence, we can conclude that our compound protects the adipocytes from abrupt lipolysis and stimulates adipocyte differentiation. In addition, it plays a hepatic protective role by shifting clearance and uptake of lipoproteins and fatty acids to the peripheral tissues and retrieving the fatty liver condition.

## 1. Introduction

Type 2 diabetes mellitus (T2DM) is a heterogeneous, chronic metabolic disorder categorized by impaired insulin secretion from pancreatic *β*-cells or by the presence of a persistent hyperglycemic and insulin resistance state. Insulin resistance is responsible for the disruption of biomolecule metabolism such as carbohydrates, protein, and fats. In T2DM, another important hallmark, which arises because of insulin resistance, is dyslipidemia [1, 2]. According to the International Diabetes Federation (IDF) Diabetes Atlas, 10th edition, currently, 537 million cases of diabetes are reported worldwide. In 2021, IDF ranked Pakistan third in having the highest number of reported cases of diabetes. According to IDF, Pakistan has 33 million reported cases of diabetes and has an outrageous comparative diabetes prevalence of 30.8%. Pakistan also ranked fourth in having the highest number of annual deaths 0.4 million due to diabetes. Of total annual deaths, 29% of the deaths are associated with diabetes [3].

Insulin binds to the heterotetrameric insulin receptor (INSR) on its extracellular *α*-subunit and, as a result, leads to conformational changes and causes the autophosphorylation of tyrosine residues. After autophosphorylation, these residues attract insulin receptor substrates (IRSs) and after binding to INSR, IRSs also phosphorylate tyrosine and lead to the cascade of downstream signaling [4]. The regulatory subunit of PI3K contains the SH2 domain which binds to IRS1/2 phosphotyrosines residues and leads to the recruitment of PI3K catalytic domain and leads to the activation of PI3K and converts PIP2 to PIP3 at the plasma membrane. AKT is activated when its PH domain binds with PIP3 and by upstream kinases such as PDK1, which phosphorylate its catalytic domain at Thr308 [5]. Insulin-mediated regulation of AKT signaling is involved in translocating GLUT4 to the membrane and upregulates the glucose uptake and also regulates the *De novo* lipogenesis (DNL) and protein synthesis [6]. In adipocytes and the liver, lipogenesis is regulated by the activation of mTORc1 through insulin-mediated PI3K/AKT downstream signaling cascade [7], which stimulates the processing and expression of sterol regulatory element binding protein (SREBP)1c [8]. SREBP1c also upregulates lipogenic genes such as fatty acid synthase (FAS), acetyl-CoA carboxylase (ACC), diacylglycerol O-acyltransferase (DGAT), and LPL and facilitates the DNL. SREBP1c/ADD-1 also regulates adipocyte differentiation by upregulating the expression of *PPARγ* either by providing the transcription factor or by directly activating it [9].

Obesity is a major factor responsible for insulin resistance and leads to T2DM; because of insulin receptor (IR), three crucial insulin-responsive tissues such as the liver, skeletal muscle, and adipose tissues become insulin insensitive [10]. Obesity leads to the accumulation of lipids in adipocytes and is responsible for triggering the release of inflammatory markers such as tumor necrosis factor-*α* (TNF*α*), interleukin-6 (IL-6), and C-reactive protein (CRP) and for insulin resistance [11, 12]. Several studies demonstrated that in the case of IR, there is an ectopic accumulation of fat in hepatic tissue, which intensifies the hepatic steatosis condition [13, 14]. Normally, insulin suppresses the lipolysis, but in the case of insulin resistance, it fails to restrain lipolysis, but the hepatic lipogenesis is still enhanced, which leads not only to hyperglycemia condition but also induces hepatic steatosis [15]. In addition to SREBP, overexpression of ChREBP in mice leads to fatty liver and steatosis conditions [16]. Hepatic PPARy overexpression leads to enhanced steatosis by elevating the expression of a protein involved in triglyceride storage, lipid droplet formation proteins (FSP27 and perilipin-1), and lipid uptake by CD36 and FATPs [17]. Studies also reported that in various cell lines, TNF*α* leads to the activation of the NF-*κ*B pathway; in consequence, there is enhanced expression of protein-tyrosine phosphatase 1B (PTP1B). Other inflammatory markers such as IL-6 and IL-1*β* also augmented the expression of PTP1B by activating the NF-*κ*B pathway [18]. PTP1B is a negative regulator of the insulin signaling pathway (ISP), which can lead to T2DM, so it is considered a major pharmacological target to cure T2DM. PTP1B can downregulate ISP by dephosphorylating the tyrosine residue of INSR and IRS-1 [19]. Studies have supported that that overexpression of PTP1B negatively affects protein tyrosine kinases [20], and in consequence, induces leptin and insulin resistance because insulin is unable to bind with INSR and finally leads to T2DM [21]. Overexpression of PTP1B has been reported in almost every tissue such as the liver, pancreas, adipose tissues, and muscle of rodents in obesity and T2DM [22].

*Dodonaea viscosa* (L.) Jacq, a medicinal plant, and an evergreen shrub belong to the family of Sapindaceae, locally familiar with the name “*pribet*” and “*Ghawraskay*.” *Dodonaea viscosa* originated from Australia but spread out the other countries such as Africa, Mexico, Virginia Island, New Zealand, Florida, India, and Pakistan [23]. Traditionally, this plant is also used to restrain hyperglycemic situations and to improve diabetes [24]. *Dodonaea viscosa's* aerial parts also have shown hypolipidemic, antioxidant, and antidiabetic activities in the streptozotocin (STZ)-induced diabetic animal model [25]. Recently, Uddin et al. reported the in vitro antidiabetic activity of viscosol compound, which is extracted from the aerial part of *Dodonaea viscosa*. Viscosol was recognized as the most potent negative regulator of PTP1B activity [26, 27]. In a previous study, the antidiabetic potential of flavonolic phytochemical viscosol scientifically named 5,7- dihydroxy-3,6-dimethoxy-2-(4-methoxy-3-(3-methyl but-2-enyl) phenyl)-4Hchromen-4-one- was evaluated in a high-fat diet and low-dose STZ-induced diabetic mice model [27]. The specific aim of the current research is the evaluation of the hypolipidemic activity of viscosol in the in vivo high-fat diet (HFD)-STZ–induced diabetic mice model. We have evaluated the insulin signaling pathway, DNL, adipocytes differentiation, augmentation of lipoproteins clearance and fatty acid uptake, lipid droplet formation, antilipolysis activity, and hepatic steatosis of PTP1B inhibition in adipocytes and hepatocytes of HFD-STZ–induced diabetic mice model. Graphical abstract of the study for inducing T2DM in mice is shown in (A), and the role of PTP1B in regulation of insulin-mediated lipoprotein clearance and lipid metabolism in adipocytes and hepatocytes is shown in the graphical abstract (B).

## 2. Materials and Methods

### 2.1. Animals

Mice (Mus Musculus, C57BL/6) were purchased from the primate facility of NIH, Islamabad, and only adult male mice of an average weight of 25–40 g and an average age of 8–12 weeks were selected for experimental purposes. We kept all these mice for a week in the primate facility of Quaid-e-Azam for acclimatization. All mice were facilitated with standard pellet food and water. Mice were kept and maintained at 27°C and 12 h of light and dark cycles throughout the experiment.

### 2.2. Groups

The mice were divided into 5 total groups. The experiment was performed in triplicate. Group 1 was labeled as a positive control group. Group 2 was the HFD-low dose STZinduced diabetic mice model (the diabetic mice group). Group 3 was an HFD-STZ compound-treated model (the compound-treated group). Group 4 was labeled as only the viscosol-treated group, and Group 5 was the HFD-STZ-metformin–treated group (Table 1).

### 2.3. Induction of Diabetes and Compound Treatment

The control group was given a single intraperitoneal injection of saline (500 μL). The fasting blood glucose level (BGL) (4–6 h) of the control group mice was measured by using an ACCU-CHEK Instant S glucometer (Roche Diagnostic, Mannheim, Germany). Repeated low doses of STZ (Bioworld, Catalog no. # 41910012-3) and HFD were used to generate the type 2 diabetic mice model. The diabetes was induced by the protocol as previously reported [27]. In the HFD-STZ–induced diabetic group, the HFD-STZ compound-treated group, and the HFD-STZ-metformin–treated treated, all mice were kept fasting overnight before the first injection and for 4–6 h for the rest of the injection. STZ (Bio plus Fine Research Chemical, CAT # 41910012-3, Bioworld) (40 mg/kg) dissolved in saline (500 *μ*L) was injected intraperitoneally for five consecutive days. Mice having fasting BGL above 250 mg/dL were considered diabetic. Furthermore, a single dose of viscosol 33 mg/kg (approx. 1 mg/mice), dissolved in 1% dimethyl sulfoxide (500 *μ*L) was injected intraperitoneally into the HFD-STZ compound-treated and viscosol-treated groups. After treatment with the compound, mice were monitored for 7 days, and fasting BGL was measured throughout the week. On the 17^th^ day, all the mice were euthanized.

### 2.4. Serum Blood Glucose Analysis

During the whole study, the fasting BGL was monitored by a commercial ACCU-CHEK Instant S (Roche Diagnostic, Mannheim, Germany), from the tail vein of the mice.

### 2.5. Blood Collection and Serum Separation

Blood was collected by cardiac puncture using a 1 mL syringe (23 G). Blood was collected in a 4 mL gel and clot activator vacutainer (Xinle). Then, the tube was centrifuged (Hermile Labortechnit GmbH Siemensstr-25 D-78564, Wehingen) for 10 min at 6000 rpm. Serum was collected from the supernatant and kept at −20°C for storage for further usage.

### 2.6. Lipid Profiling

We performed serum-based lipid profiling to evaluate the lipidemia situation in the HFD-STZ–induced compound-treated group. To estimate the total triglycerides, we performed the triglyceride assay. CHOD-PAP (cholesterol oxidase-phenol 2-aminoantipyrine peroxidase) assay was performed for the estimation of total cholesterol (TC), very low-density lipoprotein (VLDL), low-density lipoprotein (LDL), and HDL cholesterol using the commercially available kit (AMP diagnostic) and followed their recommended experimental protocols.

### 2.7. Histological Analysis by hematoxylin and eosin (H&E) Staining

For histological studies, tissues (adipose and liver) were isolated from the formalin and then dehydrated. Then, these dehydrated tissues were embedded in paraffin wax. Using microtome (KD202, China), a section (4 *μ*m) of these tissues was fixed on the slides and stained with H&E staining and oil red *o* stain. Then, using bright field microscopy (CX41, Olympus Microscope, Japan), the slides were examined to see tissue lysis, lipid droplets, and macrophage infiltration.

### 2.8. RNA Extraction, cDNA Preparation, and RT-qPCR

An RNA Invitrogen kit (Thermo Fisher Scientific, Cat No # 1218301 8A) was used to extract the RNA from the adipose and liver of all mice groups. We took 30–40 mg of the tissue and extracted the RNA by using the mentioned protocol. Then, the eluted RNA quality was checked by using the nanodrop machine (Colibri Spectrophotometer, Berthold Detection System GmbH 75,173 Pforzheim, Germany). After this, we used a cDNA synthesis kit (Thermo Scientific RivertAid First Strand cDNA Synthesis Kit) to prepare the cDNA, from the total RNA (1 *μ*g). MyGo Pro PCR system (MyGo PCR systems, IT-IS life sciences) was used to perform real-time, with SYBR Green PCR Master Mix (Thermo Fisher Scientific, CA, USA) and a set of primers to analyze our target genes (Table 2). We made 10 μL (1 μL of forward and reverse primer, 2 μL of SYBR Green, and the rest 6 *μ*L of the diluted cDNA) of the total reaction mixture in the eight well RT-PCR tubes (0.1 mL 8-tube strips). During real-time PCR, *PPiA* was chosen as a control for the data. After RT-qPCR, the relative mRNA expression (fold-change activity) of the target genes (Table 2) was calculated by using the 2−ΔΔCT method.

### 2.9. Statistical Analysis

To evaluate the significance of our data, we expressed our data as the mean ± SEM and two-way ANOVA for the comparison between the groups. For the pairwise multiple comparisons, we performed Tukey's test. Differences were considered significant at a *p* value < 0.05. All experiments were performed in triplicates.

## 3. Results

### 3.1. Lipid Profiling

**Figure 1:**
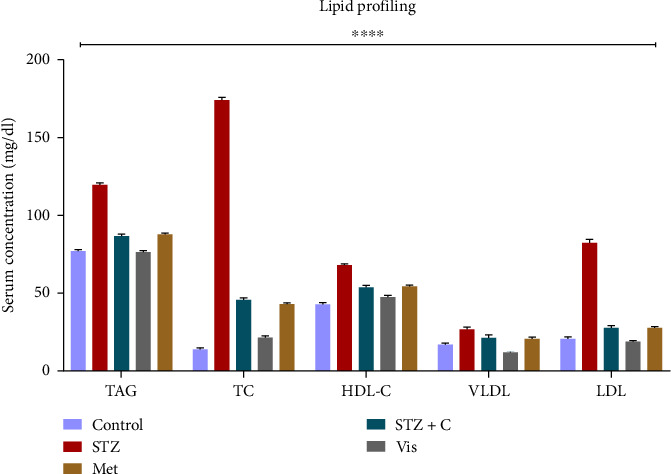
Serum lipid profiling of all experimental mice groups. Graphs represent the concentration of lipid markers in serum (mg/dL). The TAG, TC, VLDL, and LDL levels have been analyzed in the serum of all mouse groups. We found a significantly enhanced level of TAG, TC, VLDL, and LDL in our HFD-STZ–induced diabetic mice group compared with normal. We found a significant reduction of TAG, TC, VLDL, and LDL in our HFD-STZ compound-treated group. *p* < 0.0001 and *p*^∗∗∗∗^.

In diabetes, the lipid profile becomes abnormal. We performed the serum triglyceride and PAP assay. After these assays, the level of TC, VLDL-cholesterol (VLDL-C), and LDL-cholesterol (LDL-C) were measured with the available data by using the formula given previously [28, 29]. In triglyceride assay, we calculate the TAG level by the formula given in the protocol. The level of triglycerides was high in the HFD-STZ–induced diabetic mice group and a decrease in the HFD-STZ compound-treated group was observed. From the cholesterol PAP Assay, we calculated the TC. We found an elevated level of TC in our HFD-STZ–induced diabetic mice group and a decrease in TC level in the HFD-STZ compound-treated group. LDL-C and VLDL-C levels were found to be increased in the HFD-STZ–induced diabetic mice group and decreased levels in the HFD-STZ compound-treated group. In the HDL cholesterol precipitation assay, we calculated HDL cholesterol (HDL-C). This method can detect HDL-C up to 275 mg/dL. HDL-C was found to be increased in the HFD-STZ–induced diabetic mice group and decreased in the HFD-STZ compound-treated group (Figure 1).

### 3.2. Histological Analysis of Adipose and Hepatic Tissue

**Figure 2:**
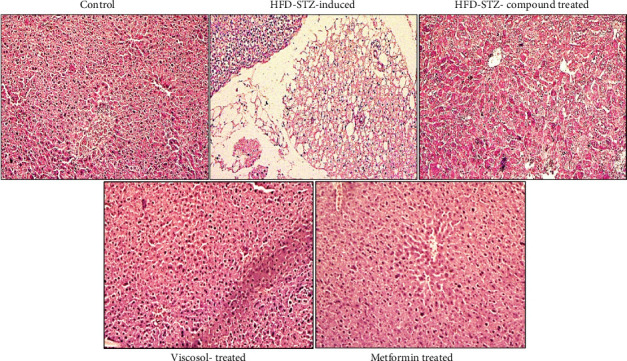
H&E staining of adipose tissue. (a) Normal morphology of the adipocytes on the simple diet, (b) increased adiposity size and lipid droplet size can be seen in the adipocytes of the HFD-STZ–induced diabetic mice group, and (c) adipocytes of the compound-treated group, showing the retrieving of the normal adipocytes morphology.

**Figure 3:**
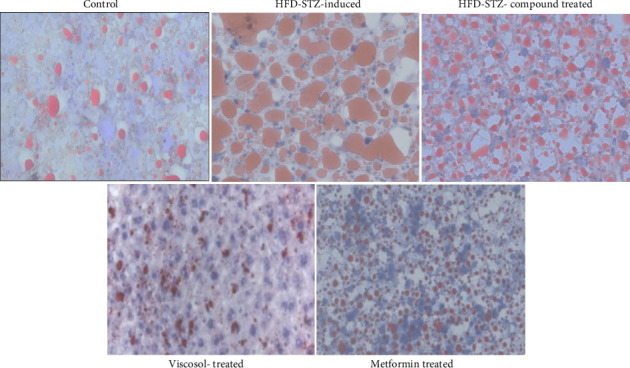
H&E staining of hepatic tissue. (a) The normal morphology of hepatocytes on the simple diet, (b) the HFD-STZ–induced diabetic mice group, seen with enormous fat buildup, a hepatic steatosis condition, and (c) the compound-treated group shows relatively similar morphology to the control.

Adipose and hepatic tissue sections were fixed on paraffin; then, tissue sections were stained by H&sE staining on the mounted slide. Then, under bright field microscopy, we analyzed the morphology of adipocytes and hepatocytes. All images were taken at 10X resolution. In adipocytes, our control group showed normal morphology, where we can see normal adipocytes and lipid droplets. But in adipocytes of the HFD-STZ–induced diabetic mice group, we observed the enlargement of the lipid droplets and increased macrophage infiltration (Figure 2). But in the HFD-STZ compound-treated group, we observed retrieval in the adipocyte's morphology and adiposity. Furthermore, in hepatocytes, we observed the same morphology both in the control and compound-treated groups. However, the lipid droplets in hepatic tissues of the HFD-STZ–induced diabetic mice group (Figure 3) with enhanced dilation of the hepatocyte cord can be seen.

### 3.3. Relative mRNA Expression of Targeted Genes in Adipocytes and Hepatocytes

#### 3.3.1. Relative mRNA Expression of Inflammatory Markers

**Figure 4:**
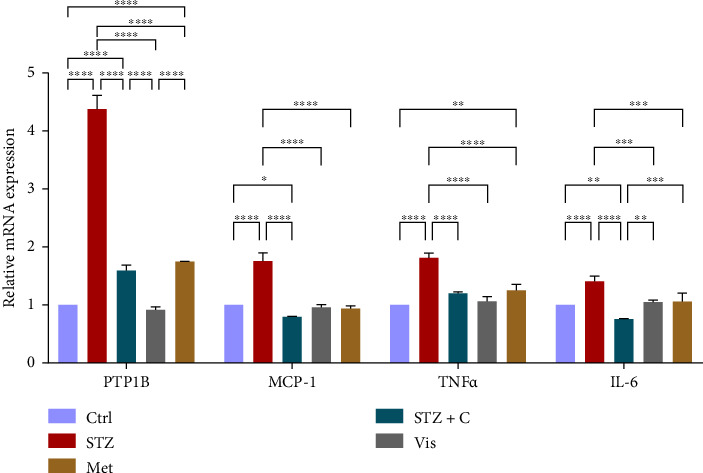
Relative mRNA expression of inflammatory markers in adipocytes shows significant variations, as depicted in the bar graph. The graphs show the relative abundance of PTP1B, MCP-1, TNF-*α*, and IL-6 as the fold change activity in the HFD-STZ compound-treated group compared with the HFD-STZ–induced diabetic mice group, taking control as the reference and comparing our compound efficacy with the standard drug metformin. We also performed the two-way ANOVA and Tukey's test to validate that our results are statistically significant, *p* < 0.05.

**Figure 5:**
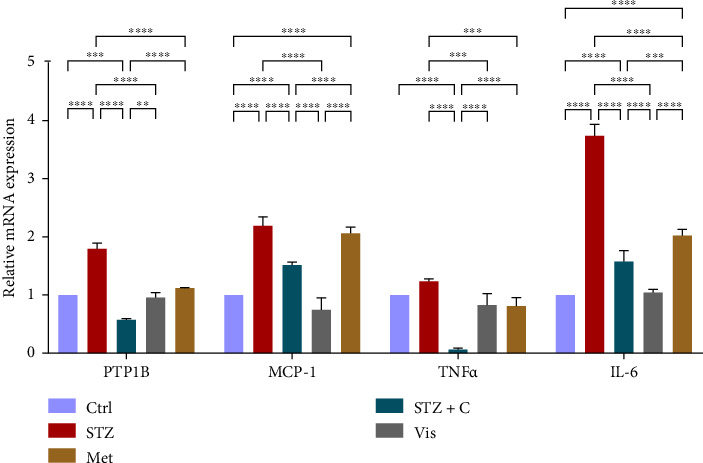
Relative mRNA expression of inflammatory markers in hepatocytes shows significant variations, as depicted in the bar graph. The graphs show the relative abundance of PTP1B, MCP-1, TNF-*α*, and IL-6 as the fold change activity in the HFD-STZ compound-treated group compared with the HFD-STZ–induced diabetic mice group, taking control as the reference and comparing our compound efficacy with the standard drug metformin. We also performed the two-way ANOVA and Tukey's test to validate that our results are statistically significant, *p* < 0.05.

PTP1B is considered the negative regulator of the insulin signaling pathway in insulin-sensitive tissues, so we targeted it to cure T2DM. For that purpose, we used the potent inhibitor of PTP1B and investigated the relative mRNA level of PTP1B and other inflammatory markers (MCP-1, TNF*α*, and IL-6) in adipocytes (Figure 4) and hepatocytes (Figure 5), which were found to be upregulated in the HFD-STZ–induced diabetic group and downregulated in our compound-treated group.

#### 3.3.2. Relative mRNA Expression of Insulin Signaling Markers

**Figure 6:**
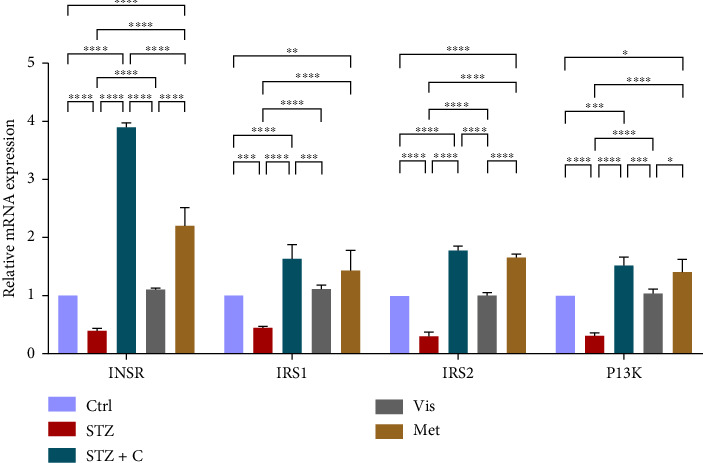
Relative mRNA expression of insulin signaling pathway markers in adipocytes has shown significant variations as can be seen in the bar graph. The graphs show the relative abundance of INSR, IRS1, IRS2, and PI3K as the fold change activity in the HFD-STZ compound-treated group compared with the HFD-STZ–induced diabetic mice group, taking control as the reference and comparing our compound efficacy with the standard drug metformin. We also performed the two-way ANOVA and Tukey's test to validate that our results are statistically significant, *p* < 0.05.

**Figure 7:**
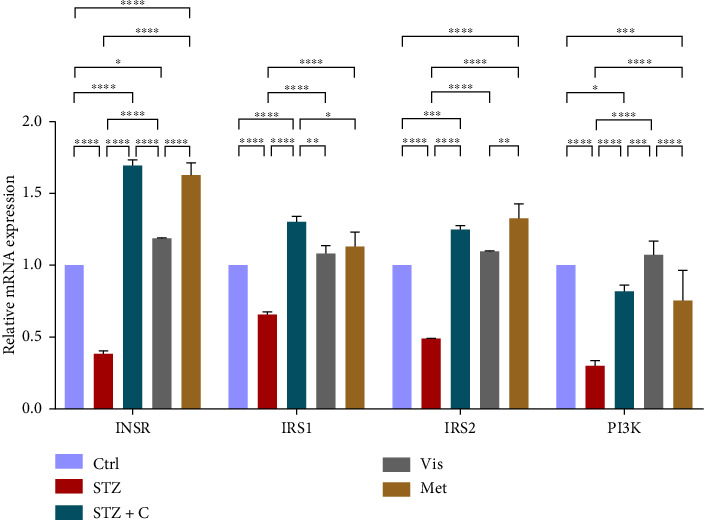
Relative mRNA expression of insulin signaling pathway markers in hepatocytes has shown significant variations as can be seen in the bar graph. The graphs show the relative abundance of INSR, IRS1, IRS2, and PI3K as the fold change activity in the HFD-STZ compound-treated group compared with the HFD-STZ–induced diabetic mice group, taking control as the reference and comparing our compound efficacy with the standard drug metformin. We also performed the two-way ANOVA and Tukey's test to validate that our results are statistically significant, *p* < 0.05.

As PTP1B regulates insulin sensitivity, we checked the relative mRNA level of the insulin signaling markers such as INSR, IRS1, IRS2, and PI3K; all of these were upregulated in the adipocytes (Figure 6) and hepatocytes (Figure 7), showing the increased expression of genes involved in insulin sensitivity in the compound-treated group.

#### 3.3.3. Relative mRNA Expression of Insulin Downstream Markers

**Figure 8:**
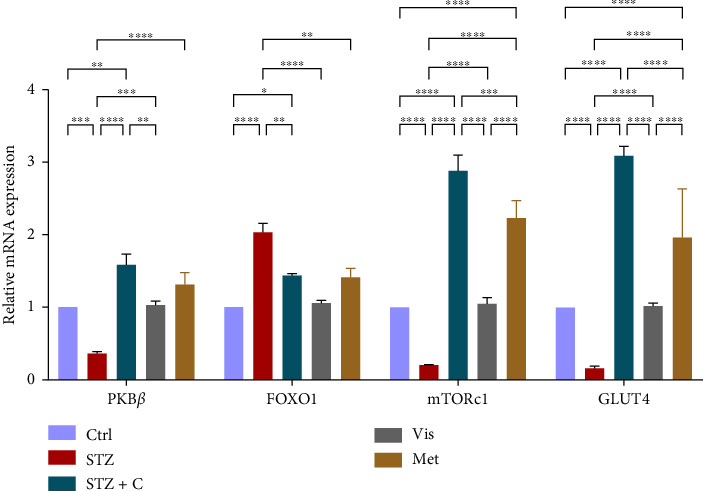
Relative mRNA expression of insulin downstream markers in adipocytes has shown significant variations as can be seen in the bar graph. The graphs show the relative abundance of PKB*β*, FOXO1, mTORc1, GLUT4, and INSIG2 as the fold change activity in the HFD-STZ compound-treated group compared with the HFD-STZ–induced diabetic mice group, taking control as the reference and comparing our compound efficacy with the standard drug metformin. We also performed the two-way ANOVA and Tukey's test to validate that our results are statistically significant, *p* < 0.05.

**Figure 9:**
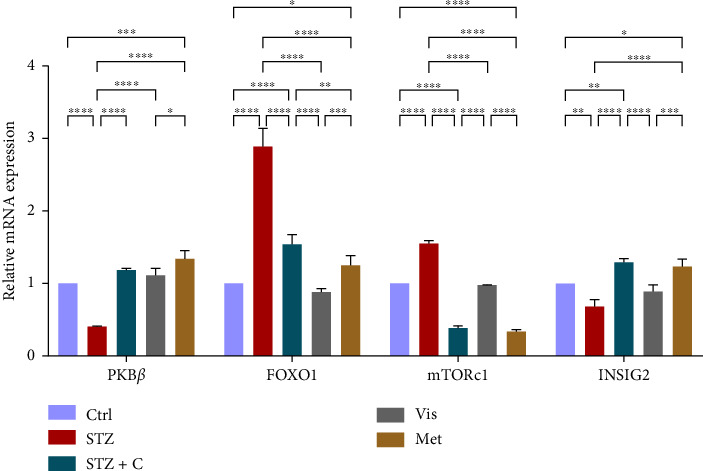
Relative mRNA expression of insulin downstream markers in hepatocytes has shown significant variations as can be seen in the bar graph. The graphs show the relative abundance of PKB*β*, FOXO1, mTORc1, GLUT4, and INSIG2 as the fold change activity in the HFD-STZ compound-treated group compared with the HFD-STZ–induced diabetic mice group, taking control as the reference and comparing our compound efficacy with the standard drug metformin. We also performed the two-way ANOVA and Tukey's test to validate that our results are statistically significant, *p* < 0.05.

In downstream signaling, insulin regulates multiple markers such as PKB*β* and FOXOa1. PKB*β* gene expression was found to be upregulated in both tissues (Figures 8 and 9) and FOXOa1 was found to be downregulated in both tissues of the compound-treated group (Figures 8 and 9). In adipocytes, mTORc1 gene expression was upregulated in the adipocytes (Figure 8) and downregulated in hepatocytes of the compound-treated group (Figure 9). In adipocytes, glucose uptake was measured by the relative mRNA expression of GLUT4, which was significantly upregulated in the compound-treated group (Figure 8). In hepatocytes, we checked the INSIG2 expression, which was found to be downregulated in the compound-treated group (Figure 9).

#### 3.3.4. Relative mRNA Expression of Lipogenic Gene Markers

**Figure 10:**
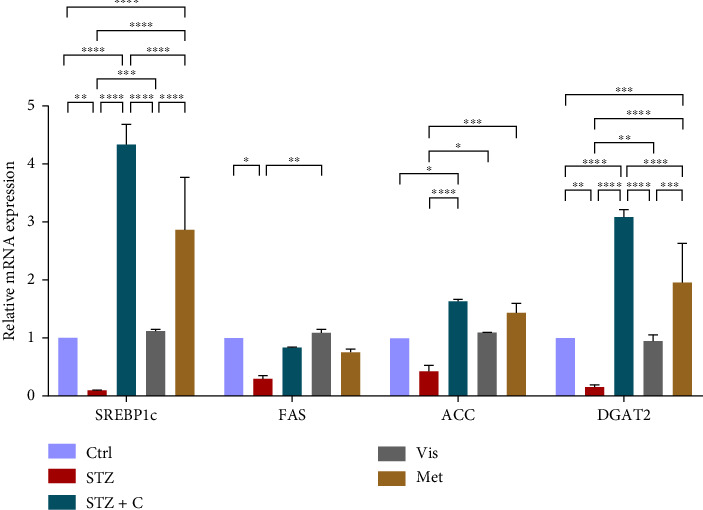
Relative mRNA expression of lipogenic gene markers in adipocytes has shown significant variations as can be seen in the bar graph. The graphs show the relative abundance of SREBP1c, FAS, ACC, and DGAT2 as the fold change activity in the HFD-STZ compound-treated group compared with the HFD-STZ–induced diabetic mice group, taking control as the reference and comparing our compound efficacy with the standard drug metformin. We also performed the two-way ANOVA and Tukey's test to validate that our results are statistically significant, *p* < 0.05.

**Figure 11:**
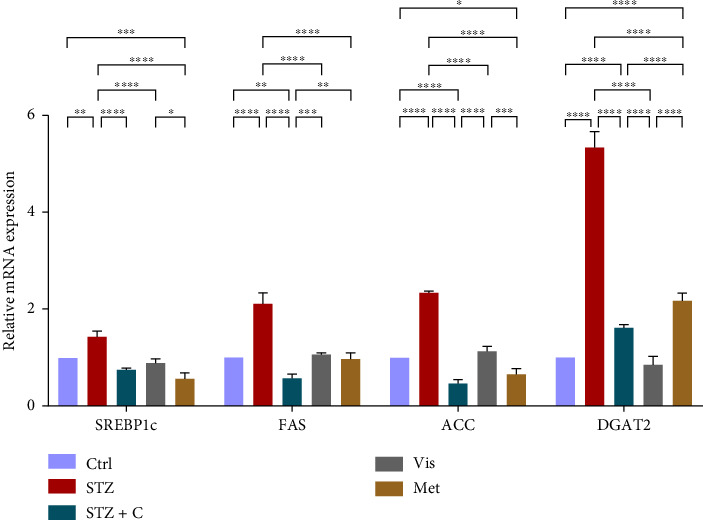
Relative mRNA expression of lipogenic gene markers in hepatocytes has shown significant variations as can be seen in the bar graph. The graphs show the relative abundance of SREBP1c, FAS, ACC, and DGAT2 as the fold change activity in the HFD-STZ compound-treated group compared with the HFD-STZ–induced diabetic mice group, taking control as the reference and comparing our compound efficacy with the standard drug metformin. We also performed the two-way ANOVA and Tukey's test to validate that our results are statistically significant, *p* < 0.05.

As lipogenic gene markers are mostly regulated by insulin signaling through the activation of mTORc1 by the PI3K/AKT signaling. mTORc1 further regulates lipogenic gene markers such as SREBP1c, FAS, ACC, and DGAT2. We checked the relative mRNA expression of all these in the adipocytes and hepatocytes. Their gene expression was found to be elevated in the adipocytes (Figure 10) and decreased in the hepatocytes (Figure 11) of the compound-treated group.

#### 3.3.5. Relative mRNA Expression of Adipocyte Differentiation, Anti-inflammatory, and Cholesterol Efflux

**Figure 12:**
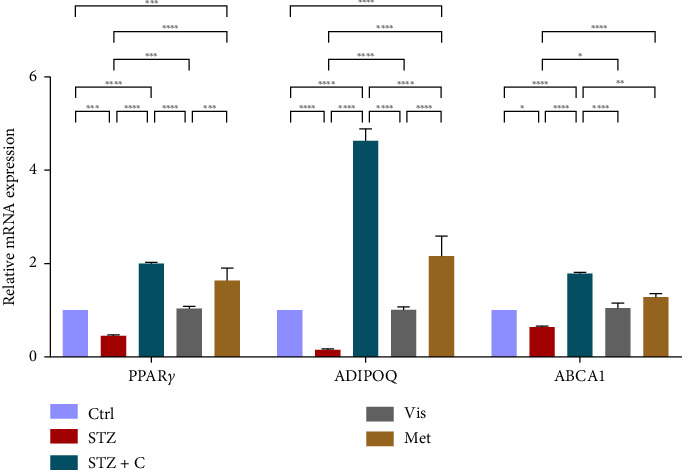
Relative mRNA expression of adipocyte differentiation, anti-inflammatory, and cholesterol efflux markers in adipocytes has shown significant variations as can be seen in the bar graph. The graphs show the relative abundance of PPAR*γ*, adiponectin, ABCA1, and ApoA1 as the fold change activity in the HFD-STZ compound-treated group compared with the HFD-STZ–induced diabetic mice group, taking control as the reference and comparing our compound efficacy with the standard drug metformin. We also performed the two-way ANOVA and Tukey's test to validate that our results are statistically significant, *p* < 0.05.

**Figure 13:**
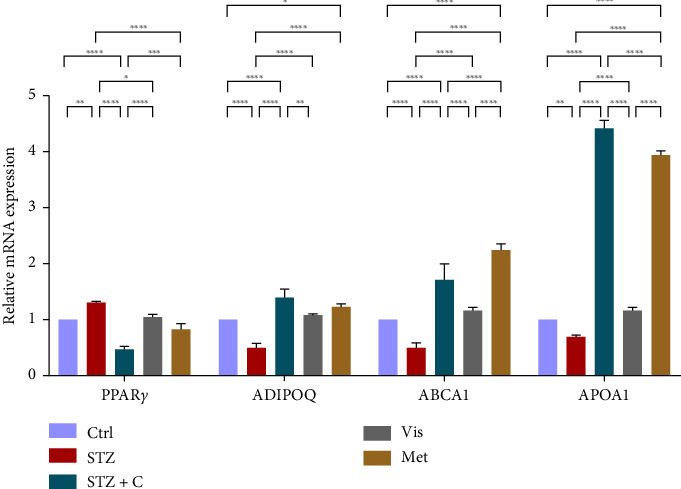
Relative mRNA expression of adipocyte differentiation, anti-inflammatory, and cholesterol efflux markers in hepatocytes has shown significant variations as can be seen in the bar graph. The graphs show the relative abundance of PPAR*γ*, adiponectin, ABCA1, and ApoA1 as the fold change activity in the HFD-STZ compound-treated group compared with the HFD-STZ–induced diabetic mice group, taking control as the reference and comparing our compound efficacy with the standard drug metformin. We also performed the two-way ANOVA and Tukey's test to validate that our results are statistically significant, *p* < 0.05.

The master regulator of adipogenesis, PPAR*γ* gene expression, was found to be upregulated in the compound-treated adipocytes (Figure 12) but downregulated in the hepatocytes (Figure 13), showing the contribution to the regulation of hepatic steatosis. AdipoQ and ABCA1 expressions showed elevated levels in both adipocytes (Figure 12) and hepatocytes (Figure 13). The ApoA1 gene is mostly expressed in hepatocytes, so its relative mRNA expression was measured and found to be upregulated in the compound-treated group (Figure 13).

#### 3.3.6. Relative mRNA Expression of Lipoprotein Clearance and Fatty Acid Uptake

**Figure 14:**
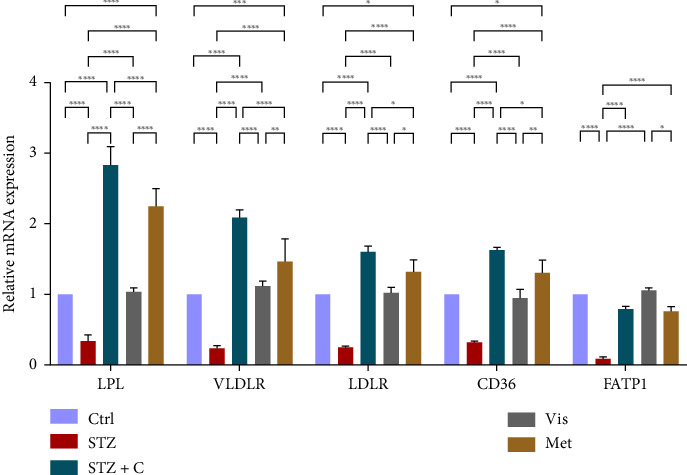
Relative mRNA expression of lipoprotein clearance and fatty acid uptake markers in adipocytes has shown significant variations as can be seen in the bar graph. The graphs show the relative abundance of LPL, VLDLR, LDLR, CD36, and FATP1 as the fold change activity in the HFD-STZ compound-treated group compared with the HFD-STZ–induced diabetic mice group, taking control as the reference and comparing our compound efficacy with the standard drug metformin. We also performed the two-way ANOVA and Tukey's test to validate that our results are statistically significant, *p* < 0.05.

**Figure 15:**
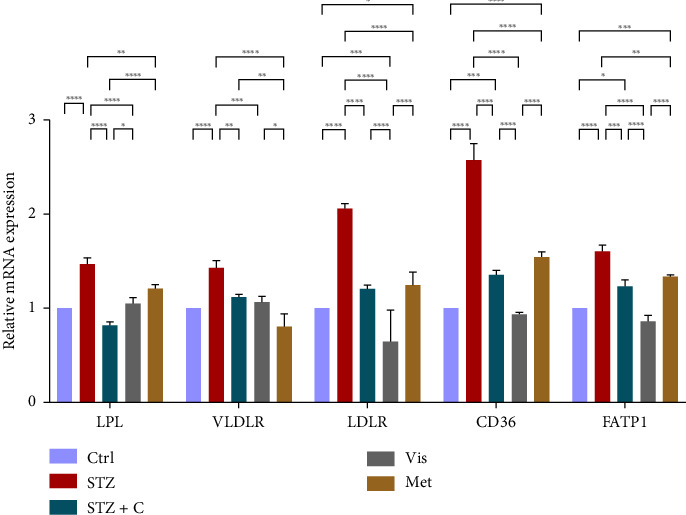
Relative mRNA expression of lipoprotein clearance and fatty acid uptake markers in hepatocytes has shown significant variations as can be seen in the bar graph. The graphs show the relative abundance of LPL, VLDLR, LDLR, CD36, and FATP1 as the fold change activity in the HFD-STZ compound-treated group compared with the HFD-STZ–induced diabetic mice group, taking control as the reference and comparing our compound efficacy with the standard drug metformin. We also performed the two-way ANOVA and Tukey's test to validate that our results are statistically significant, *p* < 0.05.

Then, we checked the relative mRNA expression of the lipoprotein clearance proteins (LPL, VLDL, and LDLR) and fatty acids uptake receptors (CD36 and FATP1) and we found that all these genes showed enhanced mRNA expression in the adipocytes of the compound-treated group (Figure 14) but found to be decreased in the hepatocytes of the compound-treated group (Figure 15) as compared with the diabetic mice group.

#### 3.3.7. Relative mRNA Expression of Lipid Droplet-Forming Proteins and Lipolytic Enzymes

**Figure 16:**
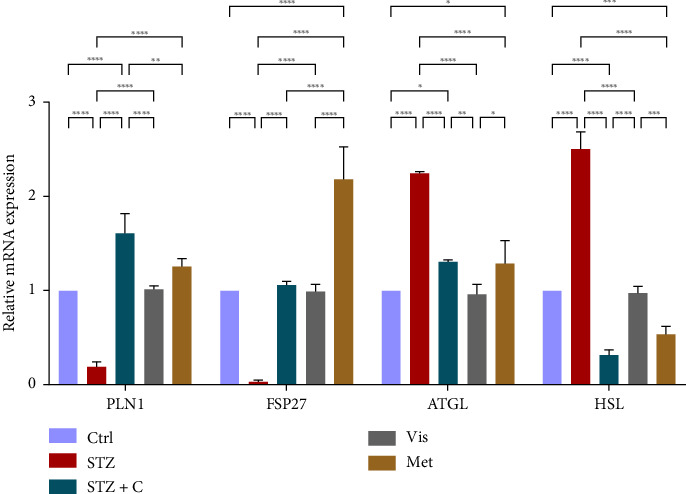
Relative mRNA expression of lipid droplet-forming proteins and lipolytic enzyme markers in adipocytes has shown significant variation as can be seen in the bar graph. The graphs show the relative abundance of Perilipin-1, FSP27, ATGL, and HSL as the fold change activity in the HFD-STZ compound-treated group compared with the HFD-STZ–induced diabetic mice group, taking control as the reference and comparing our compound efficacy with the standard drug metformin. We also performed the two-way ANOVA and Tukey's test to validate that our results are statistically significant, *p* < 0.05.

In the case of the adipocytes, we evaluated the gene expression of the lipid droplet formation protein Perilipin-1, and FSP27, which was found to be enhanced in the compound-treated group (Figure 16). Lipolysis regulating enzymes ATGL and hormone sensitive lipase (HSL) gene expression was found to be downregulated in the compound treated group as compared to the diabetic mice group (Figure 16).

#### 3.3.8. Relative mRNA Expression Lipogenic, VLDL Assembling, and Lipolytic Enzyme

**Figure 17:**
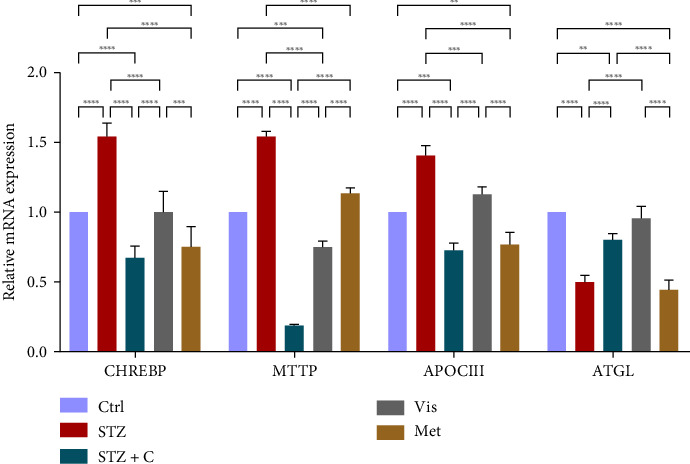
Relative mRNA expression lipogenic, VLDL assembling, and lipolytic markers in hepatocytes has shown significant variation as can be seen in the bar graph. The graphs show the relative abundance of CHREBP, MTTP, APOCIII, and ATGL as the fold change activity in the HFD-STZ compound-treated group compared with the HFD-STZ–induced diabetic mice group, taking control as the reference and comparing our compound efficacy with the standard drug metformin. We also performed the two-way ANOVA and Tukey's test to validate that our results are statistically significant, *p* < 0.05.

In hepatocytes, CHREBP is considered a secondary pathway to regulate hepatic lipogenesis and can also be responsible for hepatic steatosis in T2DM. So, we checked its gene expression which was upregulated in the diabetic mice group and downregulated in the compound-treated group (Figure 17). MTTP and APOCIII are responsible for the assembly, formation, and release of VLDL from the hepatocytes and are regulated by the FOXOa1 and can contribute to hepatic steatosis, so we checked their gene expression and we saw a decreased level of both in the hepatocytes of the compound-treated group (Figure 17). Finally, we checked the lipolytic gene ATGL gene expression in the hepatocytes of the compound-treated group and found normal lipolytic activity in the compound-treated group (Figure 17).

## 4. Discussion

T2DM is characterized by the presence of a persistent hyperglycemic and insulin resistance state. T2DM can occur because of two major possibilities, First, insulin-sensitive tissues cannot counter the insulin, and second, the impairment of *β*-cells [2]. Studies have also reported that ER stress and inflammatory cytokines are responsible for the enhanced expression of PTP1B in the liver, muscle, fat, and hypothalamus, which propagates leptin and IR [30]. PTP1B-enhanced expression in insulin-sensitive tissues has been reported in STZ-induced diabetic models of rats and mice [31]. As PTP1B is a negative regulator of the ISP, PTP1B inhibitors have been considered novel and potential target drugs for the recovery from obesity and T2DM [32, 33].

We carried out the current study to investigate the in vivo antihyperlipidemic, lipogenic, antilipolytic, adipocyte differentiation, and hepatoprotective role of PTP1B potent inhibitor viscosol (5,7-dihydroxy-3,6-dimethoxy-2-(4-methoxy-3-(3-methyl but- two-enyl) phenyl) -4H-chromen-4-one), isolated from *Dodonaea viscosa*. In vitro antidiabetic activity of viscosol has also been reported [26]. Our current study was carried out on the HFD and low-dose STZ-induced type 2 diabetic mice model, which was also reported previously [27].

Our study was started by measuring the body weight and BGL; throughout the experimental duration, we have seen a significant loss of body weight and elevation of the BGL in our HFD-low dose STZ–treated model compared to the control group, as previously reported [27]. We also performed serum-based lipid profiling, which was found to gradually decline in the HFD-STZ compound-treated model compared with the diabetic. Studies in the HFD-low dose STZ model also reported an enhanced level of serum-based lipids which was found to decrease when treated with the PTP1B inhibitor [34]. In H&E staining, we observed the enlargement of lipid droplets and enhanced macrophage infiltration in the HFD-STZ–induced diabetic mice compared with the control but no change in adiposity in our compound-treated group. The enlargement of lipid droplets in the HFD-STZ–induced group [35] and the elevation of macrophage infiltration [36] have been reported previously.

In mice adipocytes, inflammatory gene markers such as PTP1B, TNF*α,* IL6, and MCP1 were upregulated in the diabetic mice group but downregulated in our compound-treated group. Insulin sensitivity was investigated by measuring the gene expression of *INSR, IRS1, IRS2*, and *PI3K,* which were downregulated in the diabetic mice group and upregulated in the compound-treated group. PTP1B deficient models also showed enhanced IR phosphorylation and insulin sensitivity [37–39]. We further analyzed the downstream gene expression of PKB*β* (AKT-2), FOXOa1, GLUT4, and mTORc1, and found the higher expression of PKB*β*, GLUT4, and mTORc1 in our compound treatment group, compared with the diabetic mice group and reversal seen in FOXOa1. PTP1B-specific study in rat primary adipocytes shows the decreased translocation of GLUT4 [40]. mTORc1 regulated the SREBP1c processing and expression to regulate lipogenesis [8]. So, we checked the lipogenic and adipogenic gene markers such as SREBP1c, FAS, ACC, DGAT2, and PPAR*γ*, which were found to be upregulated in our compound treatment group. Lentivirus-mediated PTP1B upregulation also decreases the lipogenic gene markers such as *SREBP1c* and FAS declines adipocyte differentiation marker PPAR*γ* and decreases the lipoprotein lipase (LPL). The opposite has been seen in the PTP1B knockdown model [41]. CD36 and FATP1 majorly regulate fatty acid uptake and were seen enhanced in our compound-treated group. Studies also reported that PPAR*γ* is responsible for the regulation of FATP1, CD36, LPL, LDLR, and VLDLR in adipocytes [42]. Our study also has seen the enhanced gene expression of LDLR and VLDLR in our compound-treated group. PPAR*γ* positively regulates adiponectin [43] and ABCA1 gene expression [44]. In our compound-treated group, both were found to be upregulated. Lipid droplet proteins such as perilipin-1 and FSP27 were found to be increased in the compound-treated group. Studies have demonstrated that perilipin-1 and FSP27 are in the regulation of the PPAR*γ* [45, 46]. AKT is considered a major regulator of lipolysis in adipocytes because it can inhibit lipolysis, and mTORc1 and FOXa1 regulate the ATGL lipolytic gene [47, 48]. In our study, we found the upregulated lipolytic genes (ATGL and HSL) expression in the diabetic group but successfully retrieved the lipolytic activity in our compound-treated group.

In HFD-STZ–induced T2DM models, enhanced hepatic TG and lipid content, with enhanced liver pathological maker have been reported [49, 50]. We investigated the hepatoprotective role of the PTP1B inhibitor in our study, and we evaluated the significant decline in the inflammatory gene markers in the hepatocytes, such as PTP1B, TNF*α*, IL6, and MCP1 in our compound-treated group. Studies also reported that PTP1B deletion facilitates the protection against hepatic inflammation [51]. Evaluation of insulin sensitivity in hepatocytes has shown a significant retrieval from insulin resistance. Insulin signaling initiators such as INSR, IRS1, IRS2, PI3K, and PKB*β* gene expression were upregulated in our compound-treated group. Our study is also supported by other studies that PTP1B deletion in the liver leads to enhanced glucose uptake and elevates insulin sensitivity [52, 53]. We also found the enhanced gene expression of INSIG2 and decreased expression of mTORc1, both regulated by PKB*β*. Expression of lipogenic gene marker was downregulated in our compound-treated group. It is also reported that PTP1B deletion in the liver protects from DNL by alleviating lipogenic markers such as SREBP1c, FAS, and ACC [54]. In our study, we found the downregulated gene expression of SREBP1c, FAS, ACC, and DGAT2. We also evaluated the gene expression of CHREBP, which was upregulated in STZ but downregulated in our compound treatment group. We can hypothesize that CHREBP may be driving lipogenesis in diabetic situations. A study proved that *CHREBP* deletion significantly reduced lipogenesis [55]. In T2DM, insulin cannot restrain the activity of FOXO1 in the hepatocytes. Disinhibition of FOXOa1 leads to the upregulation of the MTTP and APOCIII genes and enhances the hepatic VLDL level [56]. Higher *APOCIII* expression and release in serum have been reported in IR and T2DM [57]. We also saw the FOXO1, MTTP, and APOCIII gene expression in hepatic tissues in the diabetic group significantly upregulated but downregulated in the compound-treated group. A significant decrease in PPAR*γ* gene expression to the basal level was seen in our compound-treated group. Overexpression of PPAR*γ* can enhance hepatic steatosis but decrease steatosis conditions reported in the disruption of the PPAR*γ* gene. PPAR*y* expression regulates gene expression of the fatty acid uptake protein CD36 and FATPs because of which, there is an enhanced FFA uptake [17]. PPAR*γ* also elevates the adiponectin expression, which upregulates the expression of ABCA1 and APOA1, which is involved in HDL-C formation [58]. We investigated the adiponectin, ABCA1, and APOA1 gene expression, which were upregulated in the compound-treated group. Furthermore, we checked the lipolytic gene marker ATGL in hepatocytes. ATGL gene expression was downregulated in the diabetic group but reached near the basal level in our compound-treated mice group. In hepatic steatosis, a study found a decreased expression of *ATGL* [59], but liver-specific overexpression of *ATGL* leads to improvement in hepatic steatosis [60].

The in vitro activity of viscosol has already been evaluated that it is a potent inhibitor of the PTP1B enzyme [26], and its in vivo activity has also been reported in our previous research paper, where we have seen the decreased protein and gene expression of PTP1B in the pancreatic cells [27]. Then, we proceeded our study to the adipocytes and hepatocytes and evaluated the gene expression of PTP1B which was found to decrease but we still need to evaluate the effect of viscosol on the protein expression in adipocytes and hepatocytes. Viscosol off-targets besides PTP1B still needed to be evaluated. In this study, we only covered the gene-based expression of inflammatory gene markers, insulin signaling genes, lipid metabolism regulatory genes, and many more. We still needed to evaluate the proteins-based regulation of all these to evaluate the viscosol effects on the insulin signaling pathway and its role in the regulation of dyslipidemia complication in type 2 diabetes mellitus. In our current study, we investigated the hepatic steatosis condition by H&E staining, but to confirm the viscosol effect on the hepatic steatosis, we needed to proceed toward the hepatic triglyceride's quantification.

## 5. Conclusion

Finally, we can conclude that our compound viscosol (5, 7-dihydroxy-3, 6-dimethoxy-2- (4-methoxy-3- (3-methyl but-2-enyl) phenyl) -4H-chrome-4-one) was found to be a negative regulator of PTP1B gene and contribute to the reversal of inflammatory and insulin resistance at the gene level in adipocytes and hepatocytes of the HFD-STZ–induced diabetic mice model. In adipocytes, our viscosol upregulated the expression of genes involved in glucose uptake, DNL, lipid droplet proteins, antilipolytic activity, adipocyte differentiation, and fatty acid uptake. It also upregulated the genes involved in lipoprotein clearance from the blood and maybe, it contributes to the retrieval of diabetic dyslipidemia. In the case of hepatocytes, we observed the hepatoprotective role of our compound. First, it reduced gene expression of the hepatic VLDL production and secretion, retrieved the hyperlipogenesis, normalized the adipocyte differentiation and lipolysis, and maybe contributed to the retrieval of the hepatic steatosis condition.

## Figures and Tables

**Table 1:** Experimental mice groups used in the current study.

Sr. No.	Groups	Names
1	Group 1	Control (Ctrl)
2	Group 2	HFD-STZ induced (STZ)
3	Group 3	HFD-STZ-compound treated (STZ + C)
4	Group 4	Viscosol treated (vis)
5	Group 5	HFD-STZ-metformin treated (met)

**Table 2:** List of primers for RT-qPCR.

Primer name	Accession number	Sequence (5′ ⟶ 3′)	Tm (°C)	Amplicon size (bp)
mPPiA-F	NM_008907	TTGGTCCGAAGTAGCCACA	60.2	88
mPPiA-R		GCCAAGCCTTTCTCGTTTC	59.9	

mmTorc1-F	NM_020009	CCAGGAGGACATTTGTTCAGA	60.1	96
mmTorc1-R		CACTGAACACAGTAGAGCCAGTG	60.0	

mSrebp1c-F	NM_011480	GGACCTTTGTCATTGGCTGT	60.0	62
mSrebp1c-R		GCATGGTCCTGATTGCTTG	60.2	

mPpar*γ*-F	NM_001308352	AGATGACAGACCTCAGGCAGAT	60.3	85
mPpar*γ*-R		TGAAGGCTCATGTCTGTCTCTG	60.6	

mFasn-F	NM_007988	GACCCACAGTAGCAGCCAAT	60.1	116
mFasn-R		AGTCTCTCCTGGACCCTCAAG	59.9	

mAcaca-F	NM_133360	CTGAGCCTTAACCTGGATTCC	60.1	117
mAcaca-R		GTCCCTTGGATGTCCACTTG	60.4	

mCd36-F	NM_001159556	TACAGAAGACCTGGGCTTGG	60.2	104
mCd36-R		CAGAAGGGTGCACAGGAGA	60.0	

mSlc27a1-F	NM_001357180	ACACATGTTGGCTGCTGTGT	60.2	115
mSlc27a1-R		GTGGATGAACCTAAGGCACAA	60.0	

mLpl-F	NM_008509	AAGCCCCACAAGTGTAGTCG	60.2	117
mLpl-R		CCTAGCAAGGGCTAACATTCC	60.1	

mAdipoQ-F	NM_009605	GAACAGTCGACACACTTTCAGC	60.0	95
mAdipoQ-R		GAACAAGTGAGTACACGTGTGGA	60.1	

mAbca1-F	NM_013454	TCAGTTCAGGGTGTGGCAT	60.1	70
mAbca1-R		GTCAGCACAAACCAACCCA	60.6	

mApoa1-F	NM_009692	TTGGCGAGAAAGCCAGAC	60.1	76
mApoa1-R		TGGGTCTTAAGCGTCTCCAG	60.4	

mApoc3-F	NM_001289755	TAGTGGGTGTCCCATGTGC	60.4	118
mApoc3-R		GGAAGAAGCACCCCAGAGA	60.3	

mMttp-F	NM_008642	ATAAGCGACAAGGCGAGAGA	60.1	82
mMttp-R		TGACTTCCAAGTGGTCTCACTG	60.3	

mIrs2-F	NM_001081212	AAATGTGACTGGAGCAGCCT	59.9	79
mIrs2-R		AAGAGAGATCCACCCATCCC	60.3	

mPlin1-F	NM_001113471	GGGCTTGACCATCAGAACC	60.5	71
mPlin1-R		TCTGCCCACGAGAAAGGA	60.5	

mCidec-F	NM_001301295	ATGGTGCCAGAGTGGTTAGC	60.1	95
mCidec-R		GGCTAGGAGGGAGTGATCAGA	60.7	

mDgat2-F	NM_026384	TTACCCACCCCTTCTAGCG	60.1	101
mDgat2-R		CTCTGCCTCTCAAGAATCCCT	60.0	

mLipe-F	NM_010719	TTATGAGTGCGCTCCGAGA	60.7	77
mLipe-R		AAGTTAAGGCACAGCCCACTC	60.7	

mPnpla2-F	NM_001163689	GCCAAGCTCCAAGTTGTCC	60.8	112
mPnpla2-R		GCAGGGTCCTATGTCCTCATT	60.3	

mInsig2-F	NM_133748	AGCAGGCCATGTGACTGAG	60.0	86
mInsig2-R		GGGAGTCAGGTTCCAGCAT	60.1	

mMlxip1-F	NM_021455	TGTCGGTCTGTTTCCTCACA	60.3	73
m Mlxip1-R		CTGCCTCTCTGCTCAGGAAC	60.3	

mLdlr-F	NM_010700	CCAAATAGGCTGTCCCAGAA	60.1	130
mLdlr-R		CCAGGACCCGGTCAGTAGTA	60.0	

mVldlr-F	NM_013703	AGTGTCTGGCATCTGGTGTG	59.7	117
mVldlr-R		TGAAGCTACCTGCCATGCT	59.6	

mFoxo1a-F	NM_019739	CTGAGAAGTGCCTGATGAGGAT	60.8	117
mFoxo1a-R		GAAAGACACACACCAAGCCAC	60.6	

## Data Availability

The data used to supporting the findings of this study are available from the corresponding author upon reasonable request.

## Nomenclature

IRS: Insulin receptor substrate
STZ: Streptozotocin
PTP1B: Protein-tyrosine phosphatase 1B
Akt: Ak strain transforming
T2DM: Type 2 diabetes mellitus
ROS: Reactive oxygen species
HFD: High-fat diet
IR: Insulin receptor
SREBP: Sterol regulatory element binding protein
ACC: Acetyl-CoA carboxylase
LDL: Low density lipoprotein
HSL: Hormone sensitive lipase
FAS: Fatty acid synthase
DGAT: Diacylglycerol O-acyltransferase
